# Immunogenicity of TNF-Inhibitors

**DOI:** 10.3389/fimmu.2020.00312

**Published:** 2020-02-26

**Authors:** Sadaf Atiqi, Femke Hooijberg, Floris C. Loeff, Theo Rispens, Gerrit J. Wolbink

**Affiliations:** ^1^Amsterdam Rheumatology and Immunology Center, Department of Rheumatology, Reade, Amsterdam, Netherlands; ^2^Department of Immunopathology, Sanquin Research and Landsteiner Laboratory Academic Medical Centre, Amsterdam, Netherlands

**Keywords:** immunogenicity, antidrug antibodies, tumor necrosis factor inhibitors, drug levels, assay

## Abstract

Tumor necrosis factor inhibitors (TNFi) have significantly improved treatment outcome of rheumatic diseases since their incorporation into treatment protocols two decades ago. Nevertheless, a substantial fraction of patients experiences either primary or secondary failure to TNFi due to ineffectiveness of the drug or adverse reactions. Secondary failure and adverse events can be related to the development of anti-drug antibodies (ADA). The earliest studies that reported ADA toward TNFi mainly used drug-sensitive assays. Retrospectively, we recognize this has led to an underestimation of the amount of ADA produced due to drug interference. Drug-tolerant ADA assays also detect ADA in the presence of drug, which has contributed to the currently reported higher incidence of ADA development. Comprehension and awareness of the assay format used for ADA detection is thus essential to interpret ADA measurements correctly. In addition, a concurrent drug level measurement is informative as it may provide insight in the extent of underestimation of ADA levels and improves understanding the clinical consequences of ADA formation. The clinical effects are dependent on the ratio between the amount of drug that is neutralized by ADA and the amount of unbound drug. Pharmacokinetic modeling might be useful in this context. The ADA response generally gives rise to high affinity IgG antibodies, but this response will differ between patients. Some patients will not reach the phase of affinity maturation while others generate an enduring high titer high affinity IgG response. This response can be transient in some patients, indicating a mechanism of tolerance induction or B-cell anergy. In this review several different aspects of the ADA response toward TNFi will be discussed. It will highlight the ADA assays, characteristics and regulation of the ADA response, impact of immunogenicity on the pharmacokinetics of TNFi, clinical implications of ADA formation, and possible mitigation strategies.

## Introduction—A Brief History of TNF Inhibitor Development

During the last decades, recombinant therapeutic proteins (biologics) have revolutionized the treatment of a wide variety of diseases. Since the demonstrated clinical effectiveness and market approval of the first recombinant therapeutic protein (insulin, 1982), which was quickly followed by the first therapeutic monoclonal antibody (OKT3, 1985), the development of these therapeutics has expanded exponentially. Currently, recombinant therapeutic proteins are the fastest-growing sector in the pharmaceutical industry with an estimated value of around 150 billion dollars. Within the field of rheumatology currently seven monoclonal antibodies, two fusion proteins, and one cytokine mimic are available that aim to meet the unmet needs of treatment with empirical medication such as methotrexate. Five of these biologics belong to the group of TNF inhibitors (TNFi) and include the monoclonal antibody-based proteins adalimumab, infliximab, golimumab, and certolizumab and the fusion-protein etanercept.

The first step toward the development of therapeutic monoclonal antibodies was set in 1975 by Kohler and Millstein who discovered how to generate monoclonal antibodies *in vitro* (1). Initially the monoclonal therapeutic antibodies were of murine origin which brought about several significant shortcomings such as the development of antidrug antibodies (ADA, termed human anti-murine antibodies or HAMA at the time) (2–4), a relatively short half-life due to weak binding to the Fc receptor (5, 6), and reduced efficacy due to poor stimulation of effector functions (6, 7). In order to overcome these drawbacks, the next generation of therapeutic monoclonal antibodies were chimeric antibodies in which the murine constant domains were replaced by their human counterpart. Although chimeric antibodies such as infliximab and rituximab (anti-CD20) are less immunogenic, they can still induce ADA formation (8, 9). With further advances in antibody engineering, humanized and fully human monoclonal antibodies became available. During the process of humanization, residual mouse-related epitopes in the variable domain are replaced by human sequences while retaining the target binding properties. Fully human antibodies can be derived from phage-display or be generated in xenogenic mice carrying the human humoral immune repertoire. Humanized and fully human monoclonal antibodies are less immunogenic and have better pharmacological properties compared to the earlier antibodies, but they still induce ADA formation (2, 9, 10).

In parallel with the advancements in antibody engineering, Brennen et al. described in 1989 that blocking of TNF inhibits the production of several important pro-inflammatory cytokines (11). This novel concept, in which TNF initiates a cascade of cytokine production, designated TNF as an interesting target for the treatment of inflammatory diseases like rheumatoid arthritis (12). Although at the time the rationale for anti-TNF therapy in rheumatoid arthritis was new and not widely accepted, several TNF-inhibitors were generated as a possible treatment for bacterial septic shock (13). After demonstrating the beneficial effect of TNFi in animal models of arthritis (14) it was shown that TNFi were also effective in patients with rheumatoid arthritis (15).

Currently, five TNFi are approved by FDA and EMA, which are infliximab, etanercept, adalimumab, golimumab, and certolizumab pegol. Adalimumab and golimumab are fully human IgG1 antibodies, infliximab is a chimeric IgG1 antibody, etanercept is a fusion-protein between a human IgG1 Fc-tail and the TNF-receptor type 2, and certolizumab pegol is a PEGylated Fab fragment of a humanized anti-TNF antibody. Despite the fact that TNFi have significantly improved the treatment of rheumatic diseases, a fraction of patients needs to discontinue treatment due to ineffectiveness or adverse reactions. Both can be the result of ADA development. The first studies that drew attention toward the immunogenicity of TNFi showed a shorter drug survival in patients after subsequent doses of TNFi (16, 17). Later it was demonstrated that most TNFi induce formation of ADA (17, 18), mostly toward the idiotype of the antibody (19–21). The reported frequencies of ADA detection and ADA titers vary between studies, which can be explained by both patient- and treatment-related factors such as genetics, type of immune response, TNFi characteristics, dosing regimen and co-medication (17). In addition, the assay format used for the assessment of ADA affects the results (22). Measurements with drug-tolerant assays have shown that the majority of patients treated with a TNFi develop ADA (22). However, not all detectable ADA result in drug levels below the therapeutic window; the clinical consequence is dependent on the relative amount of drug and ADA. Even in the presence of ADA, drug levels can be sufficiently high and contribute to clinical remission. Recently published data suggests that concentrations of around 0.1–0.5 mg/L might be sufficient to control TNF (23). The foregoing emphasizes that for the assessment of the clinical relevance of immunogenicity adequate drug level measurements are essential. In this review these different aspects of immunogenicity of TNFi will be discussed in more detail.

## Assays Used for ADA Detection

Assessing the immunogenicity of TNFi is complex, amongst others due to potential interference of the drug with the assay, variable time course of the ADA response, and variability of the antibody characteristics such as affinities and isotypes. Drug interference complicates accurate quantification of ADA and thereby the assessment of its effect on PK of the TNFi and its clinical relevance (24). Information about which assay is used and familiarity with the most important characteristics of the assay are essential to interpret ADA measurement correctly.

The most important distinction that can be made between the available assays is the extent to which the assay is sensitive to the drug in the serum (either free or bound to the ADA), i.e., the drug-tolerance of the assay. When drug is present in the serum it will form complexes with ADA. Since detection of ADA in all assay formats is based on a labeled variant of the drug, this complex formation will shield binding of the detection reagent. This phenomenon is called drug interference and it will result in underestimation of the immunogenic potential of the TNFi. Early studies that focused on the immunogenicity of TNFi used assays with very low drug-tolerance (drug-sensitive assay), thereby underestimating the levels of immunogenicity. Although current assays are often more drug-tolerant, they still are affected by the level of the TNFi to a certain extent (24).

Drug-tolerant assays can, in contrast to drug-sensitive assays, also measure ADA that are bound to the TNFi. Nevertheless, even completely drug-tolerant assays will underestimate the true amount of ADA formation since ADA-drug-complexes may be cleared from the circulation more rapidly. Using the drug-tolerant assays it was discovered that the majority of patients treated with a TNFi develop an immune response toward these therapeutic proteins (22). Although drug-tolerant assays provide a more accurate assessment of the presence of ADA compared to drug-sensitive assays, they are not necessarily more useful in clinical practice. This can be explained by the fact that drug-tolerant assays also detect ADA that would not have caused a clinical relevant decrease in drug level, while drug-sensitive assays will typically only detect ADA when drug levels are below the clinically effective threshold. Not surprisingly, the strongest associations between ADA and clinical impact were mainly established using drug-sensitive assays (24–26).

The different assay formats have been reviewed before and will be discussed only briefly in this review (24–27). Widely used formats include bridging immunoassays and antigen-binding tests (ABT). In general, both the enzyme-linked immunosorbent assay (ELISA) and the electrochemiluminescence (ECL) assay are often performed as bridging formats where (labeled) drug is used for capture as well as detection. This bridging format hinges on the multivalency of the ADA that is being detected (e.g., being able to bind at least two drug molecules). Since circulating IgG4 antibodies are largely monovalent, due to half-molecule exchange (28), ADA of the IgG4 isotype will not be appropriately detected in these bridging formats, which may result in an underestimation of the ADA response. The ABT is different in that it uses a capture ligand (generally protein A) to immobilize specific and non-specific immunoglobulins present in the sample, which is followed by specific detection of the ADA using radiolabeled (in case of the radioimmunoassay, RIA) TNFi. For all these assays, drug-tolerant formats have been developed by employing acid pretreatment which dissociates the drug-ADA complexes that may be present in the sample.

## Characterization of the Anti-Drug Antibody Response

Following antigen exposure, it may take up to a week or more for specific antibodies to become detectable in circulation (29). Initially these antibodies are expected to be of the IgM isotype. In case of proper B-cell stimulation by follicular T-helper cells, isotype switching can occur leading to the generation of antibodies of the IgG isotype. Detection of these antibodies is dependent on the sensitivity and the characteristics of the assay, which in the case of ADA are generally optimized to detect antibodies of the IgG isotype. Therefore, ADA are usually detected 2–4 weeks after the first administration (30), but may have been present at undetectable levels earlier. In the absence of proper T-cell help, ADA formation is expected to remain limited to a low titer transient IgM response with little clinical impact. Since none of the currently marketed TNFi have a mucosal route of administration, antibodies of the IgA isotype are not expected to be formed. Also, antibodies of the IgE isotype, that are associated with hypersensitivity reactions, are rarely detected (31). Previous studies demonstrated that enduring exposure to toxins leads to the formation of antibodies of the IgG4 isotype, which are associated with a tolerogenic phenotype due to the lack of Fc functionality (32). Similarly, long-term exposure to TNFi was demonstrated to result in substantial IgG4 ADA production (33).

To maintain tolerance to self, negative feedback loops are in place that prevent the generation of high affinity antibodies that recognize self-epitopes. Consistent with this concept, ADA formation to therapeutic antibodies such as the TNFi appears to be almost exclusively restricted to epitopes that are drug-specific, i.e., the idiotype. Depending on the TNFi, different drug-specific epitopes can be identified [reviewed in van Schie et al. (34)]. With the exception of etanercept, all TNFi are monoclonal antibody (-based) proteins that by definition contain complementary determining regions (CDR). These hypervariable loops form the largest part of the TNF binding region, which is unique for every antibody clone. Due to this unique amino acid sequence (e.g., not present in the natural Ig pool of the patient), and potentially aided by its natural property to prompt protein binding, the antigen-binding site forms the prime immunogenic region targeted by ADA. The TNF-receptor/Fc-tail fusion protein etanercept is unique in the sense that does not have an idiotype and only the fusion region between the domains contains non-endogenous epitopes that are potentially immunogenic, which may explain its overall low immunogenicity (35).

### Drug Neutralization by ADA

When looking at the functional impact of ADA on drug level, two types of ADA can be distinguished. These are non-neutralizing ADA (or binding antibodies, BAb), that specifically bind the drug but do not affect the drug-target interaction, and neutralizing ADA (NAb), that directly (or in close proximity) bind the pharmacologically active site of the drug thereby physically interfering with the ability of the drug to bind its target. Where BAb may indirectly decrease the drug level by increasing drug clearance via immune complex formation, NAb may have a direct negative impact on functional drug level. NAb have demonstrated to be a major safety concern for enzyme replacement therapies, where cross-reactivity to and subsequent neutralization of the endogenous counterpart has led to life-threatening side effects [reviewed in Wang et al. (36)]. However, no specific safety concerns from NAb against monoclonal antibody therapeutics, including the TNFi, have been reported.

The value of specific NAb assessment for monoclonal antibody therapeutics may be questioned. Inconsistent NAb incidences are being reported for the same drug depending on the assay used for detection, which is exemplified by the market authorization reports on biosimilars (37). Further, reporting only NAb incidences easily results in misunderstanding of NAb data. Typically, ADA-positive samples are assessed in a NAb assay. When a certain degree of neutralization is observed, the sample is considered “NAb-positive,” which is easily interpreted as “in this patient, drug is inactive because it will be neutralized by NAb.” Conversely, ADA-positive samples where no neutralization was measured in the assay would be “NAb-negative.” This may be taken to mean “this sample contains non-neutralizing antibodies.” Both interpretations might be true, but not necessarily so. To interpret NAb data it is important to realize that functional neutralization depends on the relative concentrations and affinities of the ADA, the drug, the target, and the target's respective target (the TNF receptor in case of the TNFi). Since the relative concentrations of these components is different between patients and between the various compartments where the drug may act, e.g., blood vs. tissue, it is not possible to mimic the exact level of *in vivo* neutralization in a single functional *in vitro* assay. In general, the relative concentrations of these components used in NAb assays far exceeds the natural variation seen in the patient, thereby limiting the relevance of the NAb assay outcome. As a consequence, reported NAb positivity only indicates the presence of ADA that potentially could neutralize the drug, but does not inform whether this truly happens *in vivo*. Conversely, reported NAb negativity only demonstrates that neutralization was not detectable in that particular highly artificial *in vitro* assay format, but does not definitively excludes *in vivo* neutralization. It is further important to realize that NAb assays, especially cell-based NAb assays, are often less sensitive than ADA assays. As a result, samples with only low titers may be deemed positive in the ADA assay but negative in the NAb assay, thereby (wrongfully) suggesting that non-neutralizing ADA are present (25). In case of ADA to TNFi, a more precise interpretation would be presence of neutralizing antibodies in quantities insufficient (also in relation to their affinities) to neutralize a significant amount of drug.

ADA are mainly directed to epitopes in the antigen-binding site, which is why binding of ADA to the drug interferes with TNF binding. Evidence supporting this notion has been provided by serological studies that demonstrated virtually complete (90–97%) loss of binding between ADA and TNFi in the presence of excess TNF (21), demonstrating that TNF and ADA binding are mutually excluding. Recently, this was also demonstrated for other therapeutic antibodies, for example natalizumab (38). These ADA were investigated in more detail by several studies (20, 38–40). The study by Cassotta et al. on natalizumab demonstrated by crystallography that monoclonal ADA that were scored positive or negative (i.e., below an arbitrary cut-off, weak neutralization was observed) for *in vitro* neutralizing functionality both occupied the same physical space as the drug target, suggesting that, given high enough concentrations, both types of ADA would be neutralizing (39). Taken together, these studies suggest that whenever an ADA response to any of these therapeutic antibodies (and probably any therapeutic antibody) is detected, most if not all ADA will have the capacity to neutralize. Therefore, there is no additional information to be gained from NAb testing in this setting. Together with the lack of specific safety concerns related to NAb and the lack of *in vivo* relevance and inconsistency of reported NAb assay outcomes, NAb testing for monoclonal antibody therapeutics and their biosimilars could be omitted.

## Regulation of Immunogenicity

### Covariates Influencing Immunogenicity

Previous studies have identified several patient- and treatment-related factors that influence the immunogenic potential of TNFi. It is useful to have some insight in which covariates affect the immune response toward an exogenous protein since this might help to develop strategies which potentially reduce the immunogenicity of these compounds. Treatment-related factors affecting immunogenicity are related to the structure and composition of the mAb, its use in terms of dosage, route of administration, and co-medication. The structure of a biologic will influence immunogenicity, including the primary amino acid sequence, glycosylation and other post-translational modifications. Furthermore, the formulation of the drug can impact both chemical and physical stability, such as the tendency to aggregate. For instance, a higher murine content may trigger more ADA formation, just like the presence of aggregates. The duration and dose of the treatment and the route of administration probably affect the amount of ADA that is being produced (41–43).

An important patient-related risk-factor is the genetic background of a patient. Several studies have focused on variability in HLA-type and HLA alleles have been described to be associated with ADA formation (44–46). Some HLA alleles are thought to be protective against ADA formation (HLA-DQB1^*^05, HLA-DRB1^*^01, and HLA-DRB1^*^07, with odds ratios (OR) of 0.4 95% CI [0.186–0.862], 0.25 95% CI [0.073–0.927], and 0.2895% CI [0.078–1.004], respectively), while others might increase the risk of ADA formation (HLA-DRB1^*^03 and HLA-DRB^*^011, with OR of 2.52 95% CI [1.37–4.63] and 2.64 95% CI [1.240–4.045], respectively) (45). In a recently published study performed in 1240 Crohn's disease patients from the PANTS cohort the allele HLA-DQA1^*^05, which is carried by ~40% of the European population, was also associated with a significant higher rate of ADA development [hazard ratio 1.90 (1.60–2.25)] (46). The observation that some HLA alleles are associated with an increased risk for ADA formation against multiple TNFi is intriguing and has been suggested by some as supporting evidence for the role of HLA alleles in ADA development. However, no mechanism has yet been described that functionally explains this observation. In general, studies exploring the functional association between HLA alleles and ADA formation are highly desired.

Variability in IL-10 production is another patient-related factor that might affect ADA formation (47, 48). Vultaggio et al. described that patients exposed to infliximab may initiate an adaptive cellular response resulting in the production of infliximab-specific T-cells (49). Some of these T-cells produce IL-10 which contributes to downregulation of the immune response of the infliximab-specific T-effector cells (50). When the kinetics of IL-10 and IFNγ were analyzed by Pratesi et al. it was found that the absence of IL-10 production and a low IL-10/IFNγ ratio were associated with formation of ADA (48). These data are in line with the earlier study of Bartelds et al. which described that ADA formation against adalimumab is associated with IL-10 gene polymorphisms (47). Some reservation toward these results needs to be maintained as they are based on small groups of patients. Polymorphisms in other immune response genes such as TNF and cytotoxic T-lymphocyte associated antigen 4 (CTLA-4) have been identified as risk factors toward other biological therapies (51).

Besides genetic factors, other patient-related factors that are thought to increase the risk of ADA formation are a longer disease duration, a higher baseline disease activity, and disease status. For example, patients with an activated immune system are more likely to develop ADA compared to healthy controls or immunosuppressed patients (41–43). Another risk factor that has been described is not being naïve to TNF treatment, especially when ADA can be detected toward the previous TNFi. ADA could be detected more often in patients who developed a significant ADA response toward their first TNFi compared to anti-TNF naïve patients or patients without detectable ADA toward their first TNFi (52, 53). Use of concomitant medication also affects ADA formation. If methotrexate is not used in combination with the TNFi, it is more likely that clinically relevant ADA will develop (52, 54–56). ADA formation is also affected by serum concentration of the TNFi. Sufficiently high drug levels should be present in order to dampen the immune response toward the TNFi, especially in the first three months of treatment. In the past, TNFi were administered with irregular dosing intervals which resulted in lower drug levels and a higher percentage of ADA-positive patients. One last important patient-related factor that affects ADA formation negatively is induction of immune tolerance. In some patients the production of ADA toward TNFi can decrease over time (42).

### Induction of Immune Tolerance

In some patients ADA responses are transient, which suggests a mechanism of immune tolerance. Immune tolerance refers to the absence of a measurable antibody response, skewing of the immune response to a less inflammatory phenotype, or exhaustion of the immune response to a particular immunogenic antigen. This physiological phenomenon is essential to prevent excessive immune responses to harmless antigens such as dietary antigens, allergens, and commensal microbiotics. The mechanisms contributing to immune tolerance have not yet been fully elucidated.

During treatment with TNFi, immune tolerance is mostly observed as a decrease in ADA titers over time. For example, van Schouwenburg et al. described a transient ADA response in 17/53 ADA-positive RA patients treated with adalimumab (22), while Steenholdt et al. described transient detection of ADA in inflammatory bowel disease (IBD) patients treated with infliximab (57). Two other studies also observed disappearance of ADA over time incomparable patient populations (58, 59). In a group of multiple sclerosis (MS) patients treated with natalizumab similar results have been found; anti-natalizumab antibodies develop in ~60% of patients but are persistent only in 3.5–10% of the patients (60–62).

Peripheral tolerance is likely to be of importance in acquiring immune tolerance to foreign antigens by preventing auto-reactivity. Several mechanisms have been suggested to be involved in this process, and most of them are T-cell mediated. One refers to the presentation of self-antigens by dendritic cells (DC) to T-cells. In the absence of appropriate co-stimulation, or in the presence of co-expression of ligands for the inhibiting receptorsCTLA4 and programmed cell death protein 1 (PD-1), DC fail to activate the T-cell which leads to T-cell anergy. Other mechanisms are the deletion of autoreactive T-cell clones due to repetitive activation and upregulation of Fas-ligand on the T-cell and the suppressive activity of regulatory T-cells. Development of anti-idiotypic antibodies to self-reactive antibodies is another mechanism to accomplish immune tolerance (51). Furthermore, since a large part of the antibody molecule is autologs, there may exist regulatory T cells that confer a dampening effect on an evolving immune response upon being presented peptides derived from these constant domains, possibly including parts of the IgG molecule that have been designated “Tregitopes” (63).

Hemophilia A, a bleeding disorder resulting from a deficiency in factor VIII (FVIII), is the sole disease in which the downregulation of ADA formation by induction of immune tolerance has been described (64). Consequently, it provides a model to elucidate the mechanisms involved in the induction of tolerance. Depending on the severity of the FVIII deficiency, ADA toward FVIII (called inhibitors) can be detected in 5–88% of the patients, mostly within 9–12 days after exposure (65, 66). Production of these inhibitors follows the classic immune response paradigm. Important targets for the induction of immune tolerance thus include regulatory T-cells, memory B-cells, and plasma cells producing anti-FVIII antibodies. Hausl et al. demonstrated that high levels of FVIII prevent FVIII-specific memory B-cells from differentiating into anti-FVIII producing plasma cells, and instead induced apoptosis of these cells (67). In addition, enduring exposure to FVIII in the absence of co-stimulatory pro-inflammatory signals results in the induction of regulatory T-cells. These cells produce IL-10 and TGF-beta, which inhibit the formation and activation of FVIII-specific effector T-cells, again preventing the differentiation of FVIII-specific B-cells to plasma cells. Development of anti-idiotypic antibodies against anti-FVIII antibodies have also been hypothesized to contribute to immune tolerance induction via neutralization of FVIII-specific antibodies, inhibition of FVIII-specific B-cells, and induction of apoptosis of FVIII-specific B cells (51). These concepts described are probably applicable to all antibody-based drugs, including TNFi.

Although several mechanisms which potentially contribute to induction of immune tolerance toward therapeutic proteins have been proposed, it is still largely unknown how this tolerance develops. To our knowledge, detailed studies explaining the underlying mechanisms of immune tolerance have not been performed. More insight in these processes might create opportunities to optimize treatment of therapeutic proteins, treat allergies and autoimmune diseases, and improve transplant acceptance.

## ADA Affecting the Pharmacokinetics of TNFi

### General PK of TNFi

Some familiarity with the pharmacokinetics of TNFi is essential in order to understand the clinical consequences of ADA formation toward TNFi. It was already stated that not all TNFi have the same molecular structure, which is important when evaluating their PK. The pharmacokinetic properties of therapeutic monoclonal antibodies such as infliximab, adalimumab, and golimumab have been reviewed extensively in the past (41, 42, 68–71). The most important aspects of the PK of these compounds will be highlighted in this review. In general, etanercept and certolizumab pegol have comparable PK characteristics, but relevant differences between the therapeutics will be discussed.

All approved mAbs are intravenously (iv) or subcutaneously (sc) administered immunoglobulins of the IgG family. These exogenous IgG molecules are generally eliminated by the same mechanisms as their endogenous counterparts; both target-mediated drug disposition (TMDD) and nonspecific pino- and endocytosis have been described to contribute to the nonlinear and linear elimination of mAbs, respectively, eventually leading to proteolysis of the mAb. Pino- and endocytosis result in internalization of IgG molecules by fluid endocytosis or FcγR-mediated uptake, respectively, and contribute to the linear component of mAb clearance (71). However, not all immunoglobulins will be degraded directly after they have been taken up by a cell; IgG molecules can be recycled via the neonatal Fc receptor (FcRn receptor). Once this intracellular receptor has bound to an IgG molecule it will prevent its degradation by transporting the immunoglobulin back to the cell surface. In FcRn knockout mice models IgG is eliminated 10–15 times faster, indicating that this recycling mechanism contributes to the relative long half-life of both endogenous and exogenous immunoglobulins (68). In the presence of high IgG levels saturation of the FcRn receptor has been described, resulting in nonlinear clearance. However, in general such high levels will not be reached; mAbs are administered at doses of <10 mg/kg and this will increase the total IgG level by <1–2% (71). The charge of the variable fragment of IgG antibodies also affects the clearance of mAbs via FcRn-mediated recycling. The charge can affect binding of the IgG molecule to the FcRn receptor and thus alters the half-live of the IgG molecules (72).

Binding of a mAbs to its target generally increases the clearance of a mAb nonlinearly, and this process is referred to as TMDD. In theory, higher amounts of target result in a faster clearance of mAbs. However, population pharmacokinetic modeling has not been able to demonstrate the effect of amount of target, i.e., TNF, on the clearance of TNFi. This might partly be explained by the fact that TNF levels are difficult to quantify (73), which is why disease status is regularly used as a proxy of amount of target. However, disease status is not very specific, and measurements are often subjective (74). Another possible explanation is that patients receive an excess of TNFi relative to the amount of TNF (74, 75). The effect of TMDD will only be observed when the concentration of the mAb is in the same concentration range as the target (41, 68). This is not the case for TNFi; serum concentrations of TNFi are, even at trough level, much higher than TNF levels in serum. Therefore, binding of a TNFi to its target will barely contribute to the clearance of TNFi (42, 73, 76). This is different for tocilizumab, which is a monoclonal antibody that targets the membrane-bound IL-6 receptor (IL-6R). It is the only bDMARD in which non-linear clearance has been detected. The PK of tocilizumab is mainly influenced by systemic IL-6 receptor (IL-6R) binding (76), for which CRP is commonly used as a surrogate marker (77). This can be explained by the fact that the liver can express a lot of IL-6R, resulting in a low tocilizumab concentration relative to the amount of IL-6R expression.

In contrast to target binding, binding of ADA to TNFi can alter elimination rates significantly. Increased clearance due to ADA may also be classified as target-mediated drug elimination. Ternant et al. specified that in the presence of detectable ADA, the clearance of adalimumab increases 5.5-fold at the population level (76). Berends et al. estimated an average 4-fold increase in clearance of adalimumab in the presence of detectable ADA (78). ADA detection thus seems to be one of the most important contributors to the pharmacokinetic variability seen for TNFi (42).

Compared to the pharmacokinetics of mAbs, some differences should be highlighted when evaluating TNFi with another structure. For example, certolizumab pegol is a PEGylated Fab' fragment that is derived from an anti-TNF humanized mAb. The conjugation of certolizumab to hydrophilic polyethylene glycol (PEG) chains increased the half-life of certolizumab pegol to around two weeks (76). Its clearance is somewhat different due to the absence of an Fc-tail, preventing FcRn-mediated recycling. In addition, renal excretion of the Fab' fragments has been described due to the relative small size of certolizumab (76). Certolizumab is, like the mAbs described earlier, an immunogenic molecule; anti-certolizumab antibodies can be detected in 37–65% of the patients. ADA detection is associated with lower drug levels over time, but high certolizumab levels (>10 μg/ml) could still be measured in most ADA-positive patients (79, 80).

Another bDMARD with a slightly different structure is etanercept, which is a dimeric fusion protein consisting of two p75 TNF receptors and an Fc part of an IgG molecule. Etanercept is the least immunogenic TNFi; most studies did not detect any relevant ADA toward the fusion protein (81–83). Dore et al. detected ADA toward etanercept in around 6% of the patients, but the association between ADA and PK was not investigated (84). It is therefore quite surprising that the half-life of etanercept is relatively short, with a mean ± standard deviation of 102 ± 30 h. One possible explanation is that etanercept has a lower affinity for the FcRn receptor, reducing FcRn-mediated recycling.

### Population Pharmacokinetic Modeling

Population pharmacokinetic modeling allows us to identify sources of variability in PK within study populations and also provides us with the tools to quantify these effects (41). Regarding the covariates in population PK models, the result that is quite consistent among all studies is that the detection of ADA toward a TNFi significantly increases clearance rates of the TNFi, resulting in a decrease in drug levels. This has mainly been observed for infliximab, adalimumab, golimumab, and certolizumab pegol (76). Most PK studies have included ADA detection as a dichotomous variable, but due to the highly variable nature of ADA this will probably not correctly explain all variability in clearance due to ADA. However, since continuous ADA level measurements can also be difficult to interpret, other methods to quantify the effect of ADA on clearance might be useful.

An indirect method that can be used to quantify the effect of ADA detection on clearance of therapeutic antibodies is to compare the PK of endo- and exogenous IgG antibodies. In general, the PK of these molecules is quite similar, which is reflected in clearance rates. The clearance of endogenous IgG is estimated to be 0.21 L/day, while several PK studies described a median clearance of therapeutic mAbs of 0.31 (0.066–0.535) L/day. The lowest clearance rates were described for denosumab, the highest for efalizumab, which can partly be explained by differences in their molecular structure and immunogenic potential (41). Antibody formation toward exogenous IgG molecules might explain why the clearance rates of these therapeutic proteins is higher compared to endogenous IgG molecules (41, 70). The difference in clearance rates of endo- and exogenous IgG molecules could then be used to quantify the effect of ADA formation on clearance of therapeutic proteins.

Another method that might be used to demonstrate the effect of ADA formation on serum drug levels is to compare data from patients that do and do not use concomitant methotrexate. It is known that use of concomitant methotrexate significantly increases drug levels of several TNFi (55), and this effect is probably mediated by a decrease in ADA formation. The difference in drug level between patients that do and do not use methotrexate therefore potentially reflects the effect of (undetectable) ADA formation on clearance of the TNFi.

Eventually, PK models might be able to estimate the amount of ADA that is being produced in a patient based on drug levels. If other covariates are known and corrected for, a PK model can potentially estimate the effect of ADA formation on parameters like clearance and volume of distribution. The PK of the therapeutic protein would then be used as a marker for immunogenicity.

## Clinical Implications of ADA: Impaired Response and Adverse Events

All TNFi trigger an immune response, but this response will not always have clinical implications. Usually only those patients with high ADA titers will experience a diminished clinical response or adverse events. These clinical implications will be discussed.

### Impaired Response

Previous studies have demonstrated a diminished clinical response of TNFi due to the development of ADA. Three meta-analyses have shown that the presence of ADA reduces the number of patients that reach clinical response (17, 18, 85). ADA reduce clinical efficacy by neutralizing the drug, preventing it from binding to TNF, and by enhancing the clearance rate due to formation of complexes. Many of the published studies investigating the clinical relevance of ADA used drug-sensitive assays, meaning that ADA can only be detected in the absence of drug, and such studies report strong associations with ADA and loss of response. In studies using drug-tolerant assays the association between the presence of ADA and clinical inefficacy is much weaker as ADA detection is independent of the drug concentration. This is demonstrated for RA patients treated with adalimumab, IBD patients treated with infliximab and for MS patients treated with natalizumab (25). The clinical effects of ADA formation are dependent on the amount of drug that is neutralized by ADA, and the amount of free drug present. Drug-sensitive assays as a rule will correlate with the amount of free drug, whereas drug-tolerant assays will not. This is why it is important to report the quantity of ADA within patients, next to the percentage of patients that develop ADA.

The amount of administered drug correlates with serum drug levels and these drug levels correlate with the therapeutic effect. Several studies have explored the relationship between drug level and treatment response. These studies, focusing on several different autoimmune diseases, demonstrated that good responders in general have higher drug levels compared to non-responders or moderate responders (54, 86). Interestingly, there is evidence for an upper bound above which no additional clinical benefit is achieved; Pouw et al. described a concentration-effect curve of adalimumab which demonstrated that serum drug concentrations ranging between 5–8 mg/L are sufficient for an adequate clinical response in RA (87). Similar therapeutic ranges have been reported for psoriasis and IBD (88–90). The concentration-effect curves indicate that drug levels exceeding the upper limit of the therapeutic rang do not lead to additional improvement of disease activity. Recently published data suggests that even very low drug levels (serum adalimumab concentrations around 0.1–0.5 mg/L) might be sufficient to control TNF (23). A possible explanation for the difference between the earlier mentioned therapeutic range of 5–8 mg/L and recently published data could be that higher drug levels are required during the initial phase of treatment compared to later treatment phases. This may be partly linked to the formation of a (transient) immune response in the early treatment phase. PK data from the pharmaceutical dose finding studies could help to get more insight in the relationship between drug levels and response during different treatment phases.

Even in the presence of ADA, drug levels can be sufficiently high to reach clinical remission. Clearance of the drug will be affected by these ADA since it becomes non-linear in the lower serum level ranges due to target-mediated drug disposition. Despite this decrease in half-life of the (unbound) TNFi, these lower levels could still contribute to clinical remission. Serum drug level measurements are thus crucial to get insight in the clinical relevance of ADA formation; ADA measurements cannot be interpreted correctly without the context of a drug level measurement.

### Adverse Events

Besides their ability to reduce drug levels and contribute to clinical inefficacy, the presence of ADA is also associated with adverse events. Severe reactions have been described after the formation of ADA toward therapeutic proteins resembling endogenous proteins. This is exemplified by the formation of ADA toward recombinant human erythropoietin (rhEPO). ADA against rhEPO were demonstrated to cause pure red cell aplasia due to cross-reactivity with endogenous erythropoietin (91). Although TNFi share epitopes with endogenous IgG (in case of the antibody based TNFi) and the TNF-receptor (etanercept), no cross-reactivity toward the endogenous counterparts have been described so far. However, other adverse events related to TNFi have been described.

Infusion reactions are the most common ADA related adverse event described for TNFi. They are characterized by symptoms such as fever, pruritus, bronchospasms, or cardiovascular collapse during or within the first day after drug administration (62, 92, 93). The reported incidence rate of infusion-related reactions after administration of infliximab varies between 4–15% (31). The presence of ADA to infliximab is associated with a higher risk of infusion reactions (58, 94–96). A meta-analysis by Maneiro et al. showed a 2 to 4-fold higher risk of infusion reactions in ADA-positive patients compared to ADA-negative patients (18). Approximately the same numbers were described in the meta-analysis of Thomas et al. (17). This association has also been observed for natalizumab (62) and other therapeutic monoclonal antibodies applied in oncology (97). However, the fact that the majority of patients treated with monoclonal antibodies develop ADA and only a minority of these patients develop adverse events such as infusion related reactions, indicates that ADA often do not cause an infusion reaction. Even when the presence of ADA contributes to clinical inefficacy, these ADA do often not increase the risk of developing an infusion reaction. Factors that do seem to contribute to incidence of adverse events are size and shape of TNFi-ADA complexes. Several groups have studied the size of these complexes. The majority of the complexes are dimers (one therapeutic antibody bound to one TNFi) (40). However, under specific conditions larger complexes (tetramers, hexamers, etc.) can be found as demonstrated by Rojas et al. in monkeys treated with infliximab (98) and in study of van Schie et al. in sera of patients with anti-infliximab antibodies (40). The size of these TNFi-ADA complexes depends on the ADA titer and the drug/ADA ratio. Very large complexes are formed in presence of high ADA titers and when the drug/ADA ratio is around 1:1 (40).

Multimerization of antibodies can activate the complement cascade via C1. Antibody multimers, interacting through their Fc tails and forming complexes with their Fc tail close together and pointing inwards, serve as an optimal platform for C1 to bind to and to activate complement cascade (99, 100). ADA toward therapeutic antibodies are anti-idiotypic, so ring shaped complexes are formed in which the Fc-tails point outwards instead of toward each other. However, if very large, irregularly shaped complexes are formed, Fc-tails may get closer to each other, allowing to bind C1q and activate complement cascade (40). This might explain why large and irregular shaped complexes are able to activate complement cascade. A study of van der Laken et al. (101) support this finding. In this study radiolabeled infliximab was infused in three patients with ADA. Patients with small complexes did not experience any adverse events. In contrast, one patient who had developed large complexes experienced a severe infusion reaction (101). Luckily, usually only the minority of TNFi-ADA complexes are large and irregularly shaped; these tend to be formed only at high ADA concentration. This could be due to a more rapid and effective phagocytosis and clearance of larger complexes by macrophages. Internalization of small complexes are much less efficient, which is consistent with the previous observation that dimeric complexes persist in the circulation for an extended period of time. The low incidence of severe infusion related reactions can be explained by the fact that the majority of TNFi-ADA complexes are small non-immune activating complexes (40).

Infusion related reactions can range from mild (e.g., rash, pruritus, dizziness, dyspnea) to severe (anaphylactic-like reactions such as hypotension and respiratory distress). As the latter group resembles a type 1 allergic reaction, it is thought to be IgE mediated. However, a study by van Schie et al. suggests that the majority of infusion related reactions may be IgG mediated (31). Only 11% of patients with infusion reactions had detectable IgE ADA (31). Similar results has been demonstrated in other studies (102, 103).

In addition to infusion reactions, one study describes association between thrombo-embolic events and presence of ADA to adalimumab (104). However, these findings are not validated in other studies. In this study patients with ADA had a more active disease status than patients without ADA. Thus, activation of coagulation cascade could be due to systemic inflammation instead of presence of ADA.

## Mitigation Strategies

### Concomitant Use of Immune Modulating Drugs

Immunosuppressive medication is often used in combination with TNFi to decrease ADA formation. Previous studies have demonstrated that concomitant use of immunosuppressant's reduces immunogenicity. Patients using concomitant methotrexate have lower rates of ADA development (17, 18, 85) and higher drug trough concentrations (56, 75, 105). These findings were validated in three meta-analyses. However, the reduction in risk to experience clinically relevant ADA development varies between the studies and different autoimmune diseases. The aforementioned could encourage coadministration of methotrexate in other autoimmune diseases, even when use of methotrexate is not part of the standard treatment regimen.

The mechanisms underlying the beneficial effect of methotrexate on immunogenicity and clinical response of most inflammatory diseases is still not fully elucidated. One hypothesis is that methotrexate reduces TNF levels and due to reduced target-mediated drug disposition, it contributes to higher TNFi concentrations and an improved clinical response (56, 76). However, given the high quantity of TNFi compared to TNF, a reduction in TMDD does not seem to be a plausible explanation for reduced clearance of TNFi. Like mentioned before, effect of TMDD is only noticeable when the ratio target/drug is low (64, 70, 81). A second hypothesis is that methotrexate might suppress early B- and T-cell responses leading to modulation of the immune response (69). Considering the significant role of T- and B- cells in the classical immune paradigm, this seems to be a more plausible explanation. A third hypothesis is that methotrexate reduces FcγR levels, which might result in reduced clearance of TNFi (41, 70).

Some observational studies performed in patients with inflammatory bowel disease showed that concomitant use of azathioprine and glucocorticoids reduced the incidence of ADA detection (18). However, conflicting results have been found in other studies, which is why there is still some uncertainty about the effect of these drugs regarding reducing immunogenicity.

A therapeutic protein that could theoretically interfere with the immune response toward TNFi is abatacept. Abatacept prevents T-cell activation by binding to CD80/86. Just like T-cells and costimulatory signals, CD80/86 is required for differentiation of memory B cells to plasma cells and for an effective interaction between antigen presenting cells and T-cells. Therefore, blocking one of these processes might prevent alloimmunization to therapeutic proteins. However, trials focusing on concomitant use of abatacept and TNFi did not demonstrate an improved clinical response and led to a higher infection rate (106, 107).

In hemophilia A patients with inhibitors toward FVIII, intravascular administration of immunoglobulins (IVIg) results in the suppression of inhibitors because IVIg contains anti-idiotypic antibodies toward the inhibitors (108). Combination therapy with cyclophosphamide or rituximab have also been investigated to reduce immunogenicity in hemophilia A patients. Concomitant use of IVIg and cyclophosphamide can be challenging due to technical difficulties and potential toxicity. However, concomitant rituximab, which inhibits B-lymphocytes and interferes with IgG production, seem to be promising (109, 110). To our knowledge, concomitant use of rituximab in inflammatory disease has not been studied yet. Due to an increased risk of infections, this approach might be controversial.

### Dose and Dosing Regimen

As early as in 1998, Maini et al. suggested that immunogenic tolerance can be induced by higher dosages of infliximab (111). Since then, this has been supported by several other studies that have been performedin rheumatoid arthritis and inflammatory bowel disease (17, 85). The rationale behind this is that this higher infliximab dose results in higher levels of free drug compared to the amount of drug that is neutralized by ADA. Higher infliximab doses might also accelerate ADA clearance due to formation of complexes (112). Yet, this approach is not generally applied due to the higher risk of adverse events and higher costs. Especially in the field of rheumatic diseases several alternative therapeutic proteins are available. Consequently, switching to another therapeutic protein is generally preferred over a dose increase.

In IBD the initial infliximab maintenance dosing was episodic rather than scheduled. However, soon thereafter a scheduled regimens were preferred over episodic treatment strategies, as scheduled regimens resulted in lower risk of ADA development and better response (16). Patients undergoing a scheduled treatment strategy have higher trough levels, which is associated with better response rates. Similar results has been seen in adalimumab - and certolizumab trials. Due to the higher rates of ADA formation in episodic treatment regimens, is the rate of infusion reactions higher after infliximab infusion (113–115).

The above-mentioned observations are in line with the studies that have focused on the induction of immune tolerance in hemophilia A patients. Higher levels of the therapeutic protein might inhibit the reactivation of memory B-cells and prevent them to differentiate to ADA producing plasma cells. In addition, chronic exposure to the therapeutic proteins might lead to induction of T-regulatory cells. These cells are involved in peripheral tolerance and possibly also in evolution of the immune response (67).

### Therapeutic Drug Monitoring

Considering the large variation in the natural course of inflammatory diseases and the pharmacokinetics of TNFi, a personalized treatment approach would be more appropriate than a “one size fits all” approach. Therapeutic drug monitoring (TDM) might be able to facilitate this. By combining clinical data regarding disease activity and the PK of the TNFi, the drug could be applied more efficiently. Especially in case of a diminished clinical response, TDM might be able to guide clinical decision making. For example, in RA, where TNFi have failed, two treatment approaches are available; switching to a second TNFi or switching to a therapeutic with a different mode of action. The EULAR recommendations for the management of RA advocate that any biologic agent, including a second TNF-inhibitor, can be used in case of refractory response to a previous TNFi (116). In clinical practice, switching to another therapeutic agent is often a random decision and based on clinician's preference and local preference policy. TDM could improve this process by identifying subgroups of patients who would benefit more from either a second TNFi or a non-TNFi as subsequent treatment. It has been shown that a good response to a second TNFi can be anticipated in patients that lose response to their first TNFi due to ADA formation. However, loss of clinical response to the first TNFi in the absence of ADA predicts a less effective response to a second TNFi (43, 52, 53). In this group of patients drug levels are usually sufficient to control all TNF. Therefore, TNF might not be the main cytokine provoking or perpetuating disease activity and these patients might benefit more from a switch to a biological with a different mode of action. Thus TDM, or measurement of drug levels by itself, could help to make better treatment decisions, taking immunogenicity into account.

## Conclusion

TNFi have significantly improved the management of various immunological disorders. Unfortunately, a substantial fraction of patients needs to discontinue treatment due to ineffectiveness of the drug or due to adverse reactions. Both can in part be related to the development of ADA.

Historically, the majority of studies focusing on immunogenicity used drug-sensitive assays which, in general, underestimate the amount of ADA present in the serum due to drug interference. Following the introduction of drug-tolerant assays (i.e.,: where presence of drug does not, or to a lesser extent, interfere with the measurement of ADA) it was found that many patients treated with TNFi, except for etanercept, develop ADA. Detection of ADA can be associated with a reduction in clinical efficacy, but this association is dependent on the type of ADA assay that is used. Importantly, reduction in clinical efficacy is primarily related to an inadequate drug level. ADA measurements should therefore be interpreted in the context of the assay format that was used to measure ADA and a drug level measurement. In practice, the distinction between binding and neutralizing antibodies could be omitted since almost all ADA are neutralizing. Reported lower fractions of neutralizing antibodies can in most cases be attributed to lack of sensitivity of the assay used for detection.

Several patient- and treatment-related risk factors for ADA formation are being described in this review. One example being the presence of certain high-risk HLA-alleles as an important patient-related risk factor. ADA affect the PK of the drug by increasing clearance of TNFi. This becomes clinically relevant when drug levels are decreased to the point that no longer all TNF is bound. Another potential effect of ADA formation is the development of ADA-TNFi complexes which can induce hypersensitivity reactions.

Several treatment strategies to overcome immunogenicity are already being applied in clinical practice. For example, use of concomitant methotrexate is associated with less ADA toward adalimumab and infliximab. Other mitigation strategies might be worthwhile to investigate. TDM could help to improve decision making regarding the treatment with TNFi while taking immunogenicity into account.

The principles of immunogenicity described in this review can in general also be applied to other therapeutic monoclonal antibodies with different targets.

## Author Contributions

SA, FH, and FL wrote the manuscript. TR and GW helped revise the manuscript for important intellectual content. GW supervised the manuscript.

### Conflict of Interest

The authors declare that the research was conducted in the absence of any commercial or financial relationships that could be construed as a potential conflict of interest.
